# Workplace flexibility important for part-time sick leave selection—an exploratory cross-sectional study of long-term sick listed in Norway

**DOI:** 10.1186/s12889-021-10778-w

**Published:** 2021-04-15

**Authors:** Martin Inge Standal, Odin Hjemdal, Lene Aasdahl, Vegard Stolsmo Foldal, Roar Johnsen, Egil Andreas Fors, Roger Hagen

**Affiliations:** 1 Department of Psychology, Faculty of Social and Educational Sciences, Norwegian University of Science and Technology, Trondheim, Norway; 2 Department of Public Health and Nursing, Faculty of Medicine and Health Sciences, Norwegian University of Science and Technology, Trondheim, Norway; 3 Unicare Helsefort Rehabilitation Centre, Rissa, Norway; 4 Department of Public Health and Nursing, General Practice Research Unit, Faculty of Medicine and Health Sciences, Norwegian University of Science and Technology, Trondheim, Norway; 5 Department of Psychology, Faculty of Social Sciences, University of Oslo, Oslo, Norway; 6 Research institute Modum Bad, Vikersund, Norway

**Keywords:** Workplace adjustment latitude, Psychosocial work environment, Work autonomy, Psychological resilience, Graded leave, Work activation

## Abstract

**Background:**

Part-time sick leave (PTSL) where sick-listed individuals work a percentage corresponding to their remaining work capabilities is often used to promote return to work. The effects of PTSL are uncertain due to participant selection on personal and social factors, which are not easily captured by evaluations that primarily rely on register-data. More knowledge of health-related, workplace and personal characteristics that influence the propensity to utilize PTSL is needed.

The objective of the present study was to explore whether individuals on PTSL and full-time sick leave (FTSL) differ in terms of self-reported health, workplace resources and psychological resilience while also considering known sociodemographic factors that influence PTSL selection.

**Methods:**

The study utilized a cross-sectional sample of 661 workers sick listed for 8 weeks with a 50–100% sick-listing degree. Differences between those on PTSL and FTSL with regard to current self-reported health, previous long-term sick leave, workplace adjustment latitude, psychosocial work environment, work autonomy, coping with work demands, and psychological resilience were examined and adjusted for known selection factors (age, education, gender, sector, diagnosis, and physical work) using logistic regression.

**Results:**

An inverse U-shaped curvilinear association between self-reported health and PTSL was identified. Those on PTSL also reported greater workplace adjustment latitude and better psychosocial work environment than those on FTSL. These differences persisted after adjusting for previously known selection factors. Furthermore, the PTSL group reported more work autonomy and poorer coping with work demands, but these differences were more uncertain after adjustment. The groups did not differ in terms of previous long-term sick leave or psychological resilience.

**Conclusion:**

The present study found differences between those on PTSL and FTSL with regards to self-reported health, workplace adjustment latitude and psychosocial work environment that were independent of differences identified in previous research. These results are important for future evaluations of the effect of PTSL on RTW, suggesting more attention should be paid to self-reported health status and workplace characteristics that are not captured using register data.

## Background

Long-term sick leave is costly for society and has detrimental impacts on the individual [1]. In order to reduce sickness absence rates several countries have implemented reforms promoting work-related activity for the long-term sick listed [2]. One frequently used work activity is part-time sick leave (PTSL) where individuals work a percentage corresponding to their remaining work capacity [3]. Continuing to work with sickness or disabilities (e.g., through PTSL) is believed to be beneficial for the worker as work meets important psychosocial needs and includes therapeutic elements that can be beneficial for mental health and well-being [1, 4, 5]. Furthermore, returning to the workplace before full recovery is thought to be important for the return to work (RTW) process [6, 7].

Several studies find that PTSL has favorable outcomes for RTW [8–12], but others question its overall effectiveness [13–15]. Reaching firm conclusions is difficult due to the selection effect related to the use of PTSL [16]. That is, workers who are able to utilize PTSL have different personal or workplace characteristics to workers on full-time sick leave (FTSL), and therefore different probabilities of successful RTW. For example, studies have shown that those on PTSL and FTSL differ with regards to age, gender, education, diagnosis, manual or office work, and being in the private or public sector [17–21]. Selection effects make direct comparisons between the groups difficult. There is also uncertainty around efforts to control for such confounding, for example by using propensity score matching [22]. Using a randomized controlled trial design would eliminate bias caused by selection effects but is rarely feasible when examining PTSL as these arrangements are usually national schemes and thus eligible for everyone [23]. In addition, randomizing individuals to more or less sick leave than they are capable of handling is ethically problematic. Due to these issues most evaluations of PTSL use observational register-based samples. One exception is a study by Viikari-Juntura et al. [10], in which individuals with musculoskeletal disorders were randomized to PTSL or FTSL and the PTSL group showed improved RTW outcomes. In another study, Rehwald et al. [12] included PTSL as one potential component of an intervention for newly sick-listed individuals. However, even though participation in the intervention group was randomized, the use of PTSL was decided by the administering job center, adapted for individual needs and local conditions. Thus, selection for PTSL could still play a role, for instance through work characteristics.

In previous register-based studies, most of the variability in PTSL selection seems to be explained by unobserved factors [24]. Some of these unobserved factors have been proposed to include the health of the worker, and work-related and individual characteristics which may influence the propensity to use PTSL [16, 19]. For instance, poorer health may prohibit work participation altogether [25], and is the most commonly stated reason for not working after 8 weeks of sick leave in Norway [26]. In a review of PTSL use, Kausto et al. [3] also found that practical problems at work such as low flexibility, lack of control over work arrangements and poor collaboration were the main barriers for PTSL. Factors such as self-reported health, workplace adjustments, work autonomy and social support at work are also prognostic factors for RTW and hence influence sick leave duration [25, 27–30]. Furthermore, work demands have also been shown to be a prognostic factor for RTW [28, 31]. Balancing work demands and work hours has previously been reported to be a challenge when facilitating PTSL [21, 32]. The above factors could impact both the propensity to use PTSL and the likelihood of RTW and confound the impact of PTSL on RTW.

Psychological resilience is a personal characteristic that may be relevant when examining PTSL selection. Psychological resilience consists of individual personal and social resources that help individuals adapt and function in the context of adversity or stress [33, 34]. The concept has recently gained some attention in the RTW context but more research is needed [35]. There are indications that resilience increases the functioning of those struggling with pain [36, 37], and those with chronic disease [38]. Thus, resilience could potentially also influence the propensity to remain at work despite ill health.

Differences between those on PTSL and FTSL with regards to the characteristics above are difficult to capture using registry data. Some of these factors have been examined in previous studies by self-report or using proxies, but the data are scarce and inconsistent. For instance, better health in those on PTSL has been proposed [9], while other studies report poorer health, more previous sick leave, or chronicity [3, 22, 39]. Some studies also report no differences regarding health [17, 20]. Better psychosocial work environments and less conflict at work have also been found for those on PTSL [15, 39]. However, knowledge regarding workplace adjustment latitude, work autonomy, the capacity to cope with work demands and psychological resilience is lacking.

In order to know more about the potential benefits of PTSL systems it is important to know what characterizes those who use the arrangements compared to those who do not [3, 23]. Data on how health-, work-related, and personal characteristics influence PTSL selection are scarce and inconsistent. Further knowledge of how such factors are associated with PTSL could inform stakeholders such as general practitioners (GPs), employers, RTW coordinators, and social insurance services in developing solutions for work activation. Furthermore, unobserved confounding in evaluations of PTSL could contribute to incorrect conclusions which influence recommendations and policy. More knowledge of factors associated with PTSL could thus improve work activation strategies and the accuracy of and confidence in PTSL evaluations.

The objectives of the present study were to:

1. Explore whether a sample of long-term sick listed individuals on PTSL and FTSL differ in terms of previously known selection factors of PTSL (age, gender, education, private or public sector, diagnosis, and physical work demands).
2. Explore differences between the PTSL and FTSL groups in health, workplace, and personal characteristics that could influence the propensity for PTSL (current self-reported health, previous long-term sick leave, workplace adjustment latitude, psychosocial work environment, work autonomy, coping with work demands, and psychological resilience).
3. Examine whether identified differences in objectives (1) and (2) persisted independently of the known selection factors described in objective (1).

## Methods

### Study design

This was a cross-sectional study using data from a cohort of sick listed workers in an ongoing randomized controlled trial [40]. Data in the study were collected at baseline, prior to randomization. The study was approved by the Regional Committee for Medical and Health Research Ethics in South East Norway (No: 2016/2300). Written informed consent was obtained from all participants.

### Study setting

In Norway, employees are entitled to 12 months of full wage benefits when on sick leave. The GP is usually the first point of contact for individuals seeking sick leave and PTSL is regarded as the rule rather than the exception for GPs writing sick leave certification [41]. The first 16 days of sick leave benefits are covered by the employer and the remainder is paid by the National Insurance Scheme through the Norwegian Labour and Welfare Administration (NAV) [42]. The percentage of sick leave benefits received on PTSL is proportionate to the percentage the person is sick-listed. For instance, a worker who is 70% sick-listed will receive a 70% wage replacement from NAV and 30% normal wages from his or her employer. In Norway, the employer is mainly responsible for assisting the sick-listed worker back to work. By 4 weeks of sick leave, the employer and sick-listed worker are to create a plan outlining measures which can help the worker return to work. If work related activities are not resumed within 8 weeks, justification for non-activity is required from the GP or employer documenting medical or work-related reasons respectively [42–44]. NAV has a coordinating role in sick leave follow-up, and can also suggest interventions and work activities to promote RTW, such as PTSL [45].

### Participants and recruitment

Participants in the present study were employed workers aged 18–62 at 8 weeks of current sick leave with a leave status of 50–100%. Eligible participants living in Trondheim, Central Norway, were invited to participate in the study via NAV’s electronic communication site. All participants included in the trial from August 2017 until March 2020 were included in the present study. During this period 5748 individuals were invited to participate, of which 852 (15%) accepted and received a web-based questionnaire by e-mail. This questionnaire was answered by 669 (78%) of the included participants. One participant withdrew their data from the study leaving 668 participants for the present study.

### Measurement instruments

The questionnaire included covariates that were selected for use in the present study based on previously identified differences between those on PTSL and FTSL. These variables were, age, gender, education, physical work demands, work sector, and diagnosis. Additional proposed selection factors were included based on scarce evidence of PTSL selection and based on evidence of their being prognostic RTW factors (self-reported health status, previous long-term sick leave, workplace adjustment latitude, psychosocial work environment, work autonomy, and coping with work demands) [28, 29, 31]. In addition, psychological resilience was examined due to its potential influence on the propensity to utilize PTSL.

#### Part-time sick leave

Information on sick leave degree in percentage was obtained from the sick leave certificate by NAV. This variable was dichotomized as being part-time sick listed (less than 100% sick leave percentage) or full-time sick listed.

#### Covariates—previously identified selection factors

Age was used as a continuous variable. Education was collected by asking participants to select their highest achieved educational level from seven categories: “no primary school education”, “primary school education”, “high school”, “trade school”, “three years of college or university”, “five years of college or university,” or “completed Ph.D”. Education was then categorized into primary (completed primary school education), secondary (completed high school or trade school) or tertiary education (completed a minimum of 3 years of college/university). Work sector was dichotomized as public or private and a response option “Do not know/unsure” was set to missing (*n* = 9). In Norway, the self-employed is usually viewed as belonging to the private sector. Participants were asked to describe their work using the categories “Mostly sedentary work”, “Work that demand that you walk a lot”, “Work where you walk and lift a lot”, “Heavy manual labour”, and “Do not know/unsure”. These categories were dichotomized into physically demanding or undemanding work by combining the two less demanding categories and the two more demanding categories. “Do not know / unsure” was set to missing (*n* = 20)*.* Diagnosis was obtained from NAV from the sick listing certificate. Diagnosis is usually set by the individual’s GP using the International Classification of Primary Care (ICPC-2) [46]. Diagnosis was categorized as “Musculoskeletal” (ICPC-2 L), “Psychological” (ICPC-2 P), or “Other” (containing all other diagnoses).

#### Proposed selection factors

Self-reported health was assessed using the visual analogue scale from the EQ-5D-5L questionnaire [47] where participants rate their current health on a scale from 0 to 100 (0 = worst possible health–100 = best possible health). Previous long-term sick leave was assessed by asking participants whether in their working life they previously had been on sick leave that lasted for more than 8 weeks.

Four self-developed single-item questions were used to assess workplace characteristics. Workplace adjustment latitude was examined with the question “To what degree do you feel your workplace facilitates work adjustments?”. Response options ranged from 1 (to a very low degree) to 10 (to a very high degree). Psychosocial work environment was examined by asking “How would you rate the psychosocial work environment at work? (1 is very bad and 10 is very good)”. A question querying “To what degree are you able to plan your own work? (1 is to a very small degree and 10 is to a very large degree)” was used to assess work autonomy. Coping with work demands was assessed using the question “How well do you feel you cope with the demands of your work? (1 is very badly and 10 is very well)”.

Resilience was assessed using the Resilience Scale for Adults [48, 49]. The scale consists of 33 questions with six subscales assessing the individual’s social competence, social resources, planned future, family cohesion, structured style and perception of self on a scale from 1 (low) to 7 (high). The scale is scored with a mean or sum score of the 33 items to estimate psychological resilience [50]. The mean score was used in the present study.

### Statistical analysis

To describe the sample, means and standard deviations were used for continuous variables, and counts and proportions for categorical variables. Bivariate logistic regression models were fitted to each independent variable and the dependent variable to examine whether those on PTSL and FTSL significantly differed on the variable. All variables with significant associations with PTSL in the bivariate analysis were then adjusted for the covariates to examine whether the differences between groups persisted after adjusting for previously known differences. For all logistic regression analyses, odds ratios (OR) and 95% confidence intervals (CI) were reported. Quadratic associations were also investigated for age and self-reported health as these associations with PTSL have been proposed to be curvilinear [8, 21]. F-tests were used to assess whether including the quadratic terms significantly improved the models. Observations were dropped if values for sick leave degree were missing (*n* = 7). A significance level of *α* = 0.05 was used throughout. To infer results in the regressions from missing values, 10 datasets were created using multiple imputation by chained equations. No particular patterns of missing data were identified as the most repeated pattern of missing data consisted of 3% of observations that had one missing value. Seventy-six percent of observations had complete data. Counts can be found in Table 1. All variables were used in the imputation and no auxiliary variables were used. All analyses were performed using Stata 16.1 (StataCorp. 2019. Stata Statistical Software: Release 16. College Station, TX: StataCorp LLC).

**Table 1:** Characteristics of the overall sample, part-time sick listed and full-time sick listed

Variable	Sample *n* = 661	PTSL *n* = 267 (40%)	FTSL *n* = 394 (60%)
Age (*n =* 648)	44.3 (10.0)	44.0 (9.4)	44.4 (10.4)
Gender (*n =* 660) – *n* female (%)	422 (64%)	189 (71%)	233 (59%)
Education (*n* = 659)			
Primary – *n* (%)	30 (5%)	10 (4%)	20 (5%)
Secondary – *n* (%)	207 (31%)	65 (24%)	142 (36%)
Tertiary – *n* (%)	422 (64%)	192 (72%)	230 (59%)
Physically demanding work (*n =* 635) – *n* yes (%)	220 (35%)	69 (26%)	151 (40%)
Sector (*n =* 646) – *n* private (%)	325 (50%)	121 (46%)	204 (53%)
Diagnosis (*n =* 637)			
Musculoskeletal – *n* (%)	240 (38%)	92 (35%)	148 (40%)
Psychological – *n* (%)	194 (30%)	93 (35%)	101 (27%)
Other – *n* (%)	203 (32%)	78 (30%)	125 (33%)
Self-reported health (1–100) (*n =* 597)	50.4 (21.2)	52.0 (19.4)	49.3 (22.4)
Previous long-term sickness absence (*n =* 634) – *n* yes (%)	417 (66%)	172 (67%)	245 (65%)
Workplace adjustment latitude (1–10) (*n* = 647)	6.0 (3.0)	6.5 (2.9)	5.6 (3.0)
Psychosocial work environment (1–10) (*n* = 645)	7.1 (2.6)	7.4 (2.5)	6.9 (2.7)
Work autonomy (1–10) (*n* = 642)	6.0 (2.9)	6.4 (2.8)	5.8 (3.0)
Coping with work demands (1–10) (*n* = 647)	8.0 (2.1)	7.7 (2.2)	8.1 (2.0)
Resilience (1–7) (*n* = 630)	5.1 (0.9)	5.0 (0.9)	5.1 (1.0)

*Notes*: Values given are counts (%) for categorical variables and mean (SD) for continuous variables. *Education*: Having completed primary school (primary), high school or trade school (secondary), or a minimum of 3 years of college or university (tertiary). *Physically demanding work*: Percentage of individuals that rate their work as “demanding a lot of walking and lifting” or “heavy manual labour”. *Diagnosis*: Percentage of individuals classified with musculoskeletal, psychological or ‘other’ diagnosis using the International Classification of Primary Care 2nd edition (ICPC-2). *Previous long-term sickness absence*: Percentage of individuals who reported a previous sick leave episode lasting more than 8 weeks

## Results

### Sample description

A total of 661 participants were included in the analysis. Descriptive statistics can be found in Table 1 and show that 394 participants (40%) were on PTSL. The most common part-time certificate was 50%, which was the case for nearly half of those on PTSL. The PTSL group included more women, more individuals with tertiary education, and fewer with physically demanding work and working in the private sector than the FTSL group. Inspection of work-related factors revealed that those on PTSL reported higher workplace adjustment latitude, better psychosocial work environment and more work autonomy, while also reporting poorer coping with work demands.

#### Logistic regressions**—**associations with part-time sick leave

The results of the bivariate analyses (Table 2) revealed several statistically significant differences between those on PTSL and FTSL. Individuals utilizing PTSL were more often female (OR 1.70 CI 1.22–2.37), more often had tertiary education (OR 1.81 CI 1.29–2.52), less often worked in the private sector (OR 0.72 CI 0.52–0.98), had less physically demanding work (OR 0.54 CI 0.38–0.75), and more frequently a psychological diagnosis (OR 1.48 CI 1.05–2.09). On average, individuals in the PTSL group scored higher on workplace adjustment latitude (OR 1.10 CI 1.04–1.16), psychosocial work environment (OR 1.07 CI 1.01–1.14), and work autonomy (OR 1.07 CI 1.01–1.13) but scored lower on coping with work demands (OR 0.91 CI 0.85–0.98). No linear associations were found for age, self-reported health, previous sick leave, or resilience.

**Table 2:** Logistic regression of part-time sick leave compared to full-time sick leave

Variable	Bivariate OR (95% CI)	Covariate adjusted OR (95% CI)
Age	1.00 (0.98–1.01)	0.99 (0.98–1.01)
Gender (female)	**1.70 (1.22–2.37)**	**1.57 (1.10–2.24)**
Education		
Primary	0.72 (0.33–1.56)	0.90 (0.40–2.02)
Secondary	**0.57 (0.40–0.80)**	0.73 (0.50–1.07)
Tertiary	**1.81 (1.29–2.52)**	1.39 (0.96–2.03)
Physically demanding work (yes)	**0.54 (0.38–0.75)**	**0.61 (0.42–0.89)**
Sector (private)	**0.72 (0.52–0.98)**	0.92 (0.64–1.31)
Diagnosis (ICPC-2)		
Musculoskeletal (ICPC-2 L)	0.81 (0.59–1.12)	1.03 (0.73–1.46)
Psychological (ICPC-2 P)	**1.48 (1.05–2.09)**	1.32 (0.92–1.89)
Self-reported health	1.01 (0.99–1.01)	N/A
Previous long-term sickness absence (yes)	1.14 (0.81–1.59)	N/A
Workplace adjustment latitude	**1.10 (1.04–1.16)**	**1.09 (1.03–1.16)**
Psychosocial work environment	**1.07 (1.01–1.14)**	**1.10 (1.03–1.17)**
Work autonomy	**1.07 (1.01–1.13)**	1.06 (0.99–1.12)
Coping with work demands	**0.91 (0.85–0.98)**	0.94 (0.87–1.02)
Resilience	0.93 (0.78–1.10)	N/A

*Notes*: OR and 95% CI are reported. *Education*: Having completed primary school (primary), high school or trade school (secondary), or a minimum of 3 years of college or university (tertiary). *Physically demanding work (yes)*: Rating work as “demanding a lot of walking and lifting” or “heavy manual labor”. *Diagnosis*: Diagnosis using the International Classification of Primary Care 2nd edition (ICPC-2). *Previous long-term sickness absence (yes)*: Having had a previous sick leave episode lasting more than 8 weeks. *Covariate adjusted model*: Proposed selection factors (workplace adjustment latitude, psychosocial work environment, work autonomy, coping with work demands) individually adjusted for covariates (age, gender, education, physically demanding work, sector, and diagnosis). N/A indicates that the variable was not significant in the bivariate analysis, and thus not adjusted for covariates

The covariate adjusted analyses can be found in Table 2. The statistically significant differences for gender (OR 1.57 CI 1.10–2.24) and physically demanding work (OR 0.61 CI 0.42–0.89) persisted after adjustment for the other covariates, while education, sector and diagnostic categories now revealed no significant association after adjustment. Further, the covariate adjusted analyses show that workplace adjustment latitude (OR 1.09 CI 1.03–1.16) and psychosocial work environment (OR 1.10 CI 1.03–1.17) were significantly associated with being on PTSL after adjusting for known differences. Work autonomy (OR 1.06 CI 0.99–1.12) and coping with work demands (OR 0.94 CI 0.87–1.02) were no longer significant at the 95% level.

#### Curvilinear associations

**Fig. 1:**
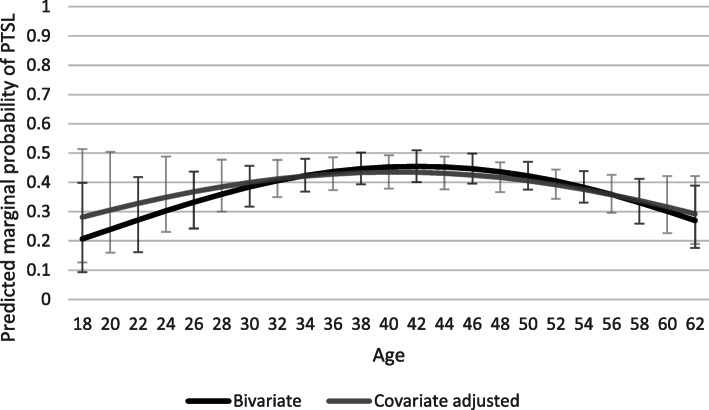
Predicted marginal probabilities of part-time sick leave by age. Unadjusted and covariate adjusted models

**Fig. 2:**
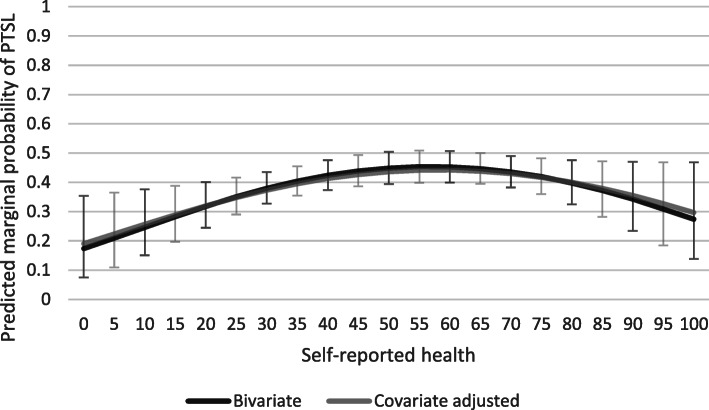
Predicted marginal probabilities of part-time sick leave by self-reported health. Unadjusted and covariate adjusted models

Curvilinear associations were found for both age and self-reported health. When adding a quadratic age term to the eqs. F-tests revealed that a quadratic age term only statistically significantly improved the bivariate model (F_1,14,218_ = 6.25, *p* < 0.012) and not the covariate adjusted model. The apex of the bivariate age-PTSL curve was at 41.9 years (see Fig. 1). When adding a quadratic self-reported health term, F-tests revealed significant improvements for the bivariate model (bivariate model: F_1,185_ = 5.75, *p* < 0.018; covariate adjusted model: F_1,211_ = 4.15, *p* < 0.043). Apexes for self-reported health were at 56.5 and 57.7 for the two curves respectively (see Fig. 2).

## Discussion

This cross-sectional study of workers sick listed for 8 weeks aimed to describe differences between those part-time sick-listed and full-time sick-listed in terms of health-, workplace-related, and personal characteristics. We also wanted to investigate whether the differences between these groups persisted after taking into account known differences from previous research.

In line with previous research the results show that individuals on PTSL were more often women, had higher education and less physically demanding work, and more often worked in the public sector [17, 19–21]. We also found less PTSL among the oldest and youngest workers, which is comparable to the findings of Ose et al. [21]. The age-PTSL curve peaked at 42 years, meaning that those in the middle of their working life were the most likely to be part-time sick listed. The present study also found more individuals sick listed due to a psychological diagnosis in the PTSL group than the FTSL group, which has also been identified in Norwegian population data [19]. After adjusting for the other covariates, however, the only significant associations on previously known selection factors were gender and physical work demands. The differences between the groups in terms of the above sociodemographic factors are usually captured in evaluations using register data. However, extra attention may need to be paid to physical work demands as information on this may not be directly available in register data and it is also a prognostic factor for RTW [31]. Furthermore, even though more individuals with a psychological diagnosis were on PTSL in the present and previous studies, it has been proposed that the potential benefits of PTSL for RTW are less convincing for workers with mental health disorders [12, 15, 51]. However, PTSL could be effective in combination with work-focused cognitive behavioral therapy [52].

We found no differences between the groups in terms of previous long-term sick leave. This supports results from Finland [17, 20], but contrasted studies which found associations with less PTSL [9] or more PTSL [22, 53]. Regarding current self-reported health we identified an inverse U-shaped curvilinear association with PTSL which largely did not change after adjustment for previously known selection factors. This association has been previously suggested due to the potential costs to the employer when facilitating PTSL [8]. Costs associated with facilitating PTSL could contribute to the curvilinear association as individuals with the best health may be close to RTW and skip PTSL altogether, while those with the poorest health may be too ill to work at all [8]. The association in the present study largely did not change after adjusting for known differences, indicating a robust curvilinear association.

The present study also found that workplace adjustment latitude and psychosocial work environment were associated with more PTSL. This could be related to more participants with higher education and sedentary work among those on PTSL, which may allow for more flexibility at work. However, adjusting for these covariates largely did not influence the associations. Workplace adjustments and psychosocial work environment also influence sick leave duration [27, 29, 30], and could confound the suggested effect of PTSL on RTW. In the present study those on PTSL also report more work autonomy and poorer coping with work demands. In previous research those on PTSL reported that the reduction in work hours was not accompanied by a corresponding reduction in productivity expectations [32], which could contribute to higher work demands for those on PTSL. However, the differences in work autonomy and coping with work demands were less convincing after adjustment which indicates that other characteristics captured by the covariates influence the relationship with PTSL (e.g., education). Finally, we found no differences between the groups with regard to psychological resilience. Resilience as measured here is a collection of psychological resources primarily relevant when faced with psychosocial adversity and may not be applicable to the diverse diagnostic sample in the current study.

Overall, there was a clear tendency that workers on PTSL had more flexible workplaces. Previous research has found that workers returning from sick leave need to have flexibility in order to successfully maintain health while meeting expected productivity demands [54]. Vooijs et al. [55] also argued that the most effective interventions to improve work participation were those focusing on changes at work rather than changing the individual’s abilities to meet work demands. However, previous research on RTW follow-up in Norway has suggested that facilitating PTSL through workplace adjustments could entail additional costs for the employer compared to hiring a substitute worker [26]. In Norway, all wages are replaced for someone sick-listed and this could contribute to a lack of incentive for the employer to facilitate adjustments [56].

Several previous studies have attempted to reduce the impact of selection when estimating the effect of PTSL on work outcomes. Markussen et al. [8] used GP propensity to certify PTSL as an instrumental variable and found that GP’s with higher propensities to certify PTSL contributed to faster RTW. Similarly, Kools and Koning [15] used Dutch insurance caseworkers’ propensity to assign PTSL as the instrumental variable, with more uncertain efficacy in terms of RTW. However, these professionals’ propensities to assign PTSL are likely also associated with their propensities to assign full RTW and possibly also their skill in facilitating RTW [15, 23]. Furthermore, PTSL possibilities also need to be negotiated with the sick-listed’s employer [15]. In this vein, Andren and Svensson [57] found an effect on RTW by using the type of occupation as an instrumental variable. This study assumed that the job type (e.g., clerks, service and sales, managers, crafts and trades) has an impact on the possibility of PTSL, but not on the possibility of full recovery from sick leave in workers with musculoskeletal illness [57]. However, it is likely that the different job types differ in work demands, which cast doubt on the assumption as work demands also influence RTW [30, 31, 58].

Others have used propensity score matching between those on PTSL and FTSL to account for selection effects, and have largely found positive impacts of PTSL on work-related outcomes [18, 20, 53]. Kausto et al. [17] also adjusted for potential confounding in multivariate analyses and found that PTSL led to reduced future disability. However, the models in these studies are based on register data and did not include variables identified in the present study, such as work flexibility or self-reported health. Bosman et al. [14], however, adjusted for such potential confounders. They included individual characteristics (age, gender and educational level), health-related factors (previous sick leave and diagnosis), and also work demands, work pace, and social support. They concluded that when adjustments were made, PTSL did not influence sick leave duration in their sample of workers with musculoskeletal disorders [14].

The present study adds to the above literature by suggesting a general tendency towards selection where sick-listed workers with flexible workplaces more frequently were on PTSL. This could mean that previous studies that adjust for differences between PTSL and FTSL using common registry data variables may not capture all important workplace characteristics that influence PTSL use. Thus, future evaluations of PTSL should consider including more detail on self-reported health and workplace characteristics to account for differences between those on PTSL and FTSL.

### Strengths and limitations

One strength of the current study is the extent of covariates which enabled us to investigate the associations between previously unexplored factors and the propensity to utilize PTSL. Registry data on the outcome variable can also be considered a strength as it helps avoid biases caused by self-report.

A limitation of the present study is the low recruitment rate of participants which could indicate participant selection. However, the present study largely found the same differences between PTSL and FTSL on the sociodemographic covariates as previous register-based studies which indicates that the current sample might be comparable to representative population studies. Another limitation in the present study is the use of single-item variables to examine workplace characteristics. Single-item variables make it difficult to determine exactly how these characteristics were different between the groups and may also lack construct validity. Future studies should use a broader set of validated questionnaires to investigate different aspects of work that could facilitate PTSL. The present study also lack data on the sick listing GP’s propensity to certify PTSL. Previous research has shown that GPs has some influence in determining sick listing percentage [59] and could thus affect PTSL selection. Finally, the present study is cross-sectional, which limits any conclusions regarding causality. For instance, the mechanisms behind poorer coping with work demands are difficult to gauge in the present study as high demands is also a risk factor for sick leave [60], and workers with manageable work demands may not be sick listed at all. Furthermore, mediation rather than confounding could explain the associations between the variables and PTSL. Further longitudinal research should thus investigate the causal pathways and potential mediation between work characteristics, covariates, PTSL, and RTW.

## Conclusions

There was significant selection for the use of PTSL in the present study. We identified differences between workers on PTSL and FTSL in terms of sociodemographic factors that were in line with previous population-based studies. The study also contributes to the existing evidence by presenting an inverse-U shaped curvilinear association between self-reported health and PTSL that needs to be further examined. Furthermore, the study indicated that those on PTSL and FTSL differ with respect to workplace flexibility independently of previously identified sociodemographic selection factors. In order to improve the accuracy of future effect evaluations of PTSL on RTW, further attention should be paid to workplace- and health characteristics that also influence RTW and are currently not captured by registries.

## Data Availability

The datasets generated and analyzed in the current study are not publicly available to protect the anonymity of participants.

## Abbreviations

FTSL: Full-time sick leave
GP: General practitioner
ICPC: International Classification of Primary Care
NAV: Norwegian Labour and Welfare Administration
PTSL: Part-time sick leave
RTW: Return to work
